# Percutaneous Coronary Intervention (PCI)-Related Coronary Artery Perforation Permanently Sealed by a Severed Inflated Balloon After Shaft Fracture: A Case Report

**DOI:** 10.7759/cureus.60295

**Published:** 2024-05-14

**Authors:** Lukáš Urban, Milan Dragula, Luboš John, Miloš Kňazeje

**Affiliations:** 1 Cardiology, University Hospital Martin, Martin, SVK; 2 Cardiology, Jessenius Faculty of Medicine in Martin, Comenius University in Bratislava, Martin, SVK; 3 Genetics, Institute of Medical Biology, Genetics and Clinical Genetics, Faculty of Medicine, Comenius University in Bratislava, Bratislava, SVK

**Keywords:** case report, percutaneous coronary intervention, acute coronary syndrome, tamponade, balloon shaft fracture, coronary artery perforation

## Abstract

Percutaneous coronary intervention (PCI) is an essential modality for the treatment of coronary artery disease. However, rare complications, such as coronary artery perforation and equipment failure, pose significant challenges. This case report describes a unique case of PCI-related coronary artery perforation and a cascade of subsequent complications managed successfully by an unconventional approach. We present a case of an 86-year-old patient who underwent coronary angiography for unstable angina and was treated with implantation of two drug-eluting stents into his right coronary artery (RCA). Implantation of the second stent caused an Ellis grade III perforation. The attempt to seal the perforation with two covered stents failed, the leak persisted, and a balloon had to be reinflated in proximal RCA. However, the patient descending into obstructive shock abruptly flexed his upper extremities breaking off the inflated balloon in proximal RCA, effectively sealing the perforation. Successful pericardiocentesis with drainage of 250 ml of blood stabilized the patient's condition and he regained consciousness. Despite moderate-intensity chest pain and extensive consultation with members of the heart team, the patient refused cardiac surgery opting for a conservative approach. The patient was discharged on post-PCI day 7, eventually resumed a physically active lifestyle, and returned for frequent follow-up visits. This case highlights the challenges in managing rare PCI complications like coronary artery perforation and balloon shaft fracture. It emphasizes the importance of rapid recognition, discusses individual techniques for the management of these complications, and focuses on the value of shared decision-making.

## Introduction

Percutaneous coronary intervention (PCI) is widely regarded as a cornerstone of invasive management of coronary artery disease [1]. The overall safety profile of contemporary PCI procedures is excellent, but rare complications including coronary artery perforation and equipment failure can lead to potentially catastrophic consequences [2]. We present a remarkable case of a patient who suffered a rapid cascade of complications stemming from an Ellis grade III perforation of a coronary artery, progressing into tamponade, obstructive shock, and a balloon shaft fracture leaving an inflated balloon in the proximal segment of a coronary artery. We further discuss individual techniques for the management of these complications.

## Case presentation

**Figure 1:**
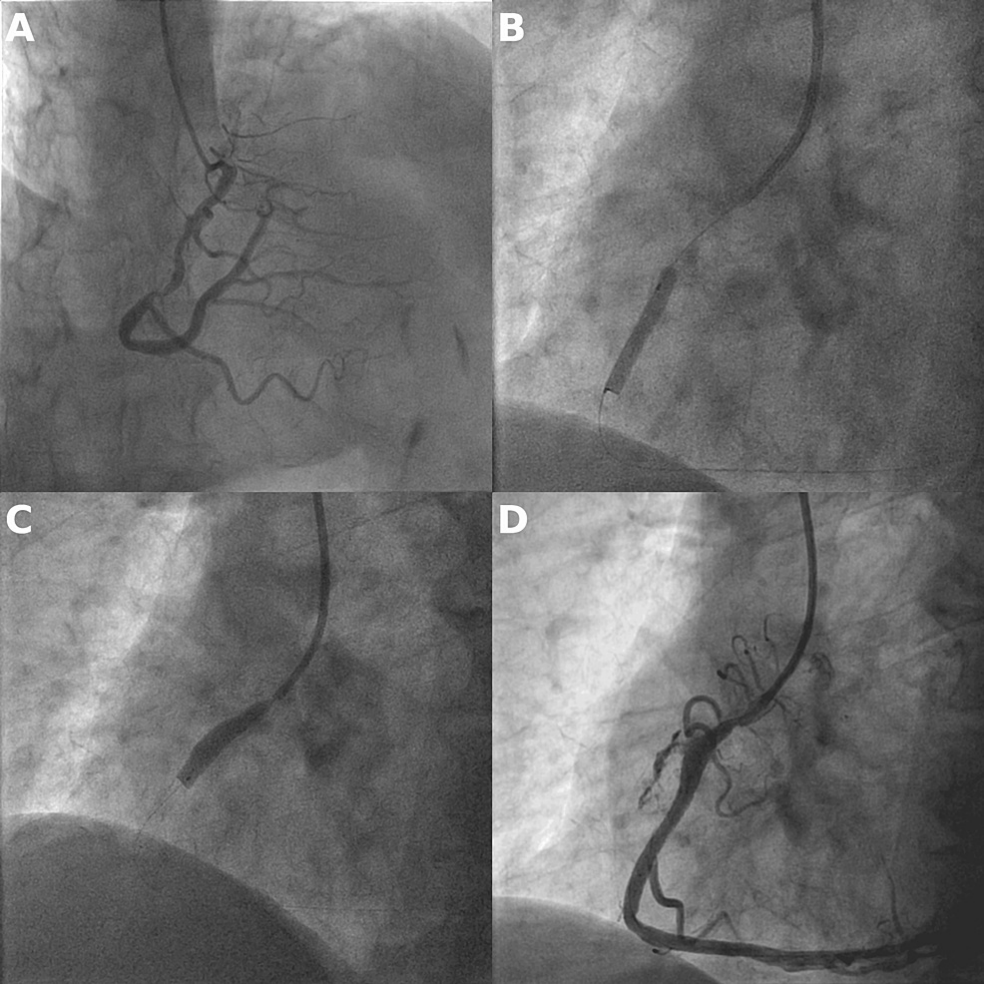
A) significant stenosis in the RCA, B) deployment of the distal 4.5 x 22 mm DES, C) deployment of the proximal 5 x 18 mm DES with visible bulging toward the side of the eventual perforation, D) Ellis grade III perforation

An 86-year-old patient was admitted to our hospital due to unstable angina. He was a non-smoker leading a physically active lifestyle; his past medical history was significant for arterial hypertension and dyslipidemia. Initial echocardiographic evaluation revealed a normal function of both left and right ventricles, no wall motion abnormalities, and no significant valvulopathy. A coronary angiography was performed with findings of marginal atherosclerotic disease of the left anterior descending (LAD) and left circumflex (LCX) arteries. A significant lesion was noted in the proximal and middle segment of the right coronary artery (RCA) (Figure 1). A decision was made to proceed with coronary intervention. The operator selected a 6 French Judkins right 4 guide catheter, wired the right coronary artery with a workhorse guidewire, and predilated the lesion with a 3 x 20 mm compliant balloon. A 4.5 x 22 mm drug-eluting stent was implanted in the distal part of the lesion, and a second 5 x 18 mm drug-eluting stent was implanted in the proximal part. Contrast injection after the second stent implantation revealed a dramatic picture of Ellis grade III coronary artery perforation with an extensive contrast extravasation into the pericardial cavity (Figure 1).

**Figure 2:**
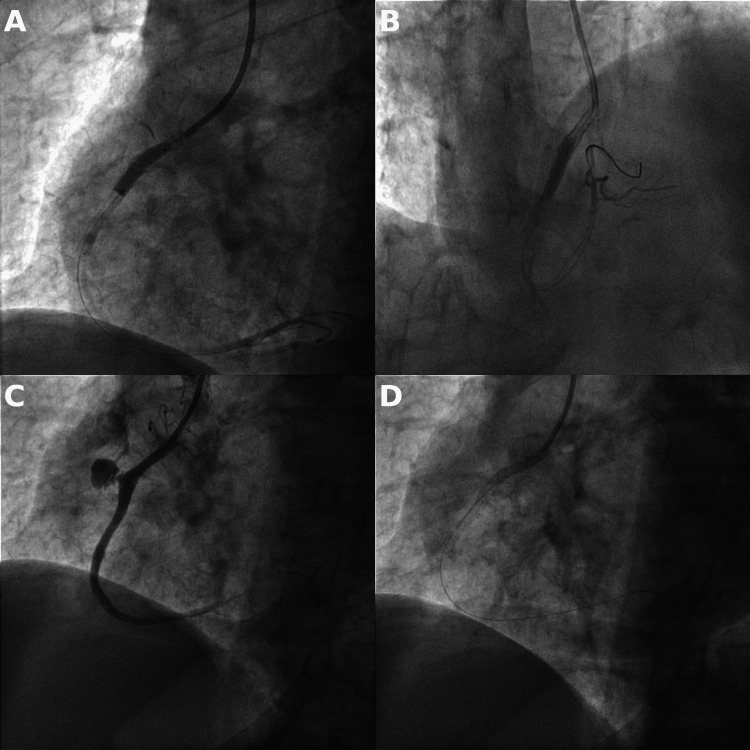
A, B) deployment of proximal and distal 3.5 x 15 mm covered stents, C) angiography displaying inadequately sealed perforation, D) fluoroscopy with visible double line of compressed right ventricle inside the blood-filled pericardium (tamponade)

The stent balloon was immediately reinflated interrupting the blood flow to the coronary artery. Two PK Papyrus 3.5 x 15 mm covered stents were subsequently deployed into the area of the vessel wall rupture. This size was chosen because at the time, unfortunately, our catheterization laboratory lacked covered stents with larger diameters. The two 3.5 x 15 mm PK Papyrus covered stents were dilated to their maximal diameter of 4.65 mm, but their deployment failed to seal the perforation and the leak continued. The formation of a double silhouette caused by the pericardium filling with a large amount of blood was apparent on fluoroscopy (Figure 2), and the patient's hemodynamic status worsened rapidly. 

**Figure 3:**
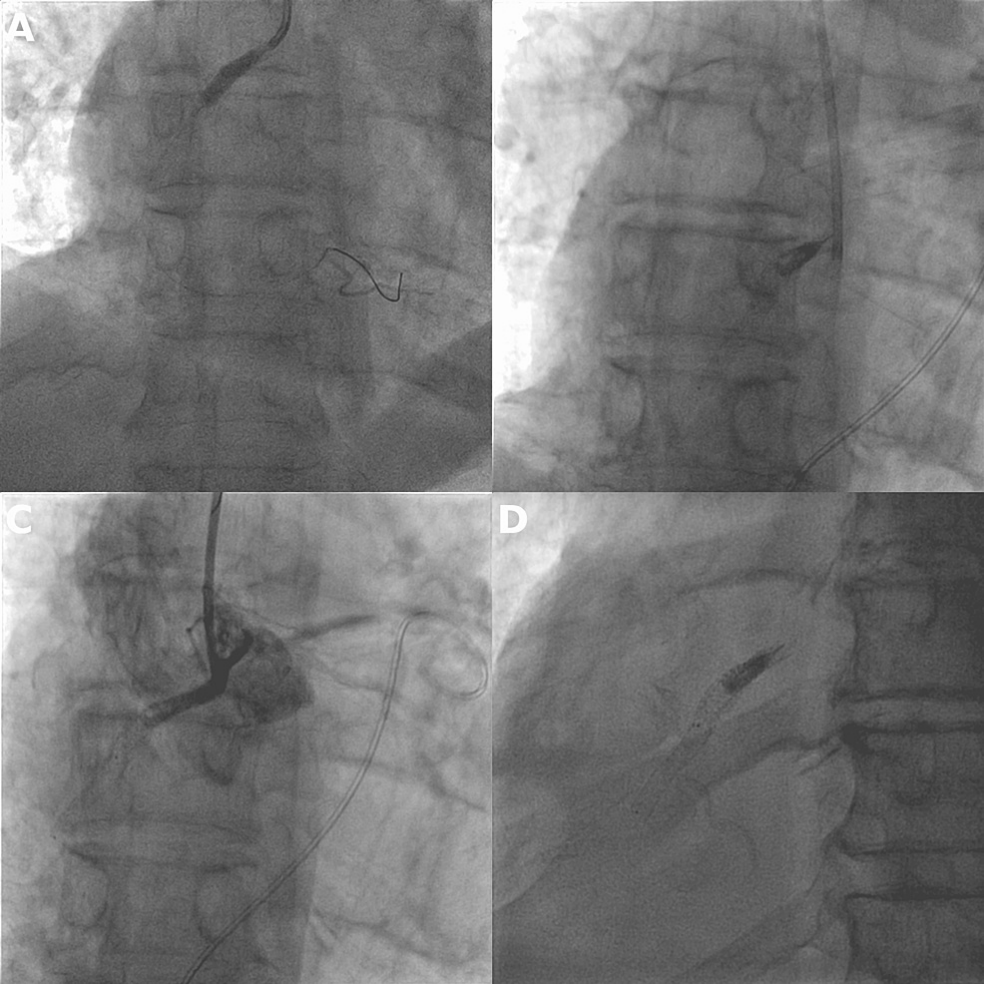
A) inflation of a 4.5 x 18 mm non-compliant balloon in the proximal part of RCA, B) detached, still inflated balloon in the proximal part of the RCA, C) angiography confirming complete occlusion of the RCA, pigtail catheter inserted in the pericardium, D) fluoroscopy one year after the index PCI with a still inflated balloon, visible double line of undersized stent graft inside the original 5 x 18 mm stent

After the last unsuccessful post-dilatation with a 4.5 x 18 mm non-compliant balloon, the balloon was reinflated in the proximal right coronary artery as preparations were made for an emergency pericardiocentesis. During the procedure, the patient´s status deteriorated into obstructive shock accompanied by a loss of consciousness and a forceful flexion of both upper extremities. A pericardial puncture was successfully performed through subxiphoid access and a 6F pigtail catheter was inserted, which drained approximately 250 ml of blood. The patient's hemodynamic status improved rapidly, and he regained consciousness. However, subsequent fluoroscopy revealed an alarming image of a catheter disengaged from the ostium of the right coronary artery, loss of the position of the guidewire, and a broken-off, still inflated 4.5 x 18 mm non-compliant balloon remaining in the proximal RCA. Angiography confirmed that the balloon fully occluded the vessel, and the leak along with the entirety of the blood flow to the right coronary artery was blocked (Figure 3).

Echocardiography (ECG) manifested ST segment elevations in the inferior leads, and the now fully conscious patient complained of moderate-intensity chest pain. A multidisciplinary team deliberated on the next course of action including possible bailout cardiac surgery. Considering the patient's firm refusal of the open chest procedure, a consensus was reached on a conservative management approach. The patient was transferred in a stable hemodynamic condition to the coronary ICU. The pigtail catheter inserted in the pericardial cavity drained only a minimal amount of blood over the next 24 hours. Repeated fluoroscopy on post-PCI days 1 and 3 displayed stationary findings of the balloon inflated in the proximal RCA. ECG revealed akinesis of the inferior wall and left ventricular ejection fraction of 48%. The right ventricular diameter 1 was enlarged to 44 millimeters, and a moderate tricuspid regurgitation was present. Additional signs of right ventricular dysfunction included reduced tricuspid annular plane systolic excursion (TAPSE) of 11 mm, paradoxical septal motion, and dilatation of both the right atrium and inferior vena cava. A guideline-directed medical therapy for heart failure was initiated, and the patient was discharged on post-PCI day 7. After discharge, the patient gradually resumed his active life and returned for periodical visits at our clinic. The balloon remained inflated and in place on follow-up fluoroscopy after three months and one year (Figure 3).

## Discussion

Coronary artery perforation and equipment malfunction are serious complications of PCI with potentially fatal consequences. Coronary artery perforation occurs in less than 0.5% of PCIs [3,4]. The incidence of perforation increases with the use of atherectomy, aggressive post-dilatation, or balloon rupture. Another possible cause of a perforation is the implantation of an oversized stent [5], as was most probably the case in our patient. Successful management of Ellis grade III perforation requires rapid recognition and occlusion of the perforation site to avert an onset of tamponade due to blood accumulation in the pericardial cavity. The first step is the reinflation of a balloon proximally or at the site of perforation. Prolonged balloon inflation can be sufficient to seal perforations, particularly if the perforation is less severe. Treatment of a large vessel perforation usually requires a stent-graft implantation [6]. A “ping-pong” technique with the introduction of a second guide catheter can be used to deliver a covered stent while a balloon remains inflated at the perforation site via the original guide catheter [7].

In our case, undersized covered stents failed to close the perforation even after post-dilatation to their maximal rated diameter. The progressing tamponade resulted in a rapid hemodynamic downward spiral ending in an obstructive shock. This state had a side effect of an abrupt flexure of the upper extremities by the patient. Swift pericardiocentesis with placement of a pericardial drain fortunately stabilized the patient; however, the patient’s forceful arm movements resulted in the detachment of the inflated balloon in the RCA. A fracture of a shaft leading to balloon detachment is exceedingly rare. To our knowledge, the rate of its occurrence is not clearly established in the literature. Possible extraction methods depend on whether the detached part of the shaft is still within the guide catheter. If so, the balloon shaft can be trapped with another balloon followed by removal of both the guide catheter and the balloon fragment. An alternative option is using a specialized snare, which is especially useful if the balloon shaft is outside the guide catheter [6].

Our case was further complicated by the fact that the balloon remained inflated even after the severing of the shaft. If the shaft was still connected, an undeflatable balloon could be managed by advancing a coronary guidewire into the balloon inflation port or an attempt could be made to burst the balloon with ultra-high pressure inflation. If the shaft is disconnected, the balloon can be perforated with a stiff CTO guidewire or the back end of a guidewire delivered through a guide extension [6]. In our case, we had to take into consideration that the deflation and removal of the balloon could potentially resume the leak into the pericardium. Lastly, the detached balloon, like any foreign object inside the heart, can predispose to thrombus formation or development of an infectious nidus [6]. For these reasons, the patient was kept on a prolonged DAPT regimen and was advised to follow infectious endocarditis prevention recommendations used in patients with artificial valves.

Cardiac surgery would be able to solve both detached balloon and perforation; however, the risk of this procedure in an 86-year-old recently hemodynamically unstable patient would be considerable. The final decision for the conservative approach was made after the patient's refusal to undergo cardiac surgery. The conservative approach resulted in a good clinical outcome and highlights the importance of shared decision-making.

## Conclusions

This report reviews a case of a PCI complicated by a unique combination of two rare complications. Coronary artery perforation is a life-threatening complication that requires immediate action to prevent hemodynamic collapse resulting from tamponade. Balloon shaft fracture with detachment of a still inflated balloon is very rare, with only a few cases described cases in the literature. Possible solutions include bursting the balloon with subsequent transcatheter extraction, surgical extraction of a still-inflated balloon, or a conservative approach in select cases.
